# Supplementary material for: Using mHealth Technology to Evaluate Daily Symptom Burden among Adult Survivors of Childhood Cancer: A Feasibility Study
Source: Cancers (Basel). 2024 Aug 27;16(17):2984. doi: 10.3390/cancers16172984 (PMC11394214; doi:10.3390/cancers16172984)
Supplement: Supplementary file 1 [file cancers-16-02984-s001.zip › cancers-3148730-supplementary.pdf]

Supplemental Material

Supplemental Table S1. Participant Participation Rate and Adherence Rate

	N (%)
Invited	60
Enrolled	41 (68)
Responded ≥3 Symptoms in Week 1	39 (95)
Responded ≥3 Symptoms in Week 5	37 (90)
Responded ≥3 Symptoms in Week 9	38 (93)
Responded ≥3 Symptoms in Weeks 1, 5 and 9	34 (83)
Responded HRQOL Week 1	41 (100)
Responded HRQOL Week 5	39 (95)
Responded HRQOL Week 9	39 (95)
Responded HRQOL in Weeks 1, 5 and 9	39 (95)
Responded ≥4 reports (≥3 symptoms and 1 HRQOL) in Week 1	39 (95)
Responded ≥4 reports (≥3 symptoms and 1 HRQOL) in Week 5	36 (88)
Responded ≥4 reports (≥3 symptoms and 1 HRQOL) in Week 9	38 (93)
Responded ≥12 reports (≥3 symptoms and 1 HRQOL) in Weeks 1, 5 and 9	34 (83)

Supplemental Table S2. Symptom variability by cancer diagnosis

**Diagnosis group**	**Variance Component**	**Variance**	**Variance (%)**	**Total Variance**
Hematological	Person-to-person	0.96	77.8%	1.23
	Month-to-month	0.06	5.2%	
	Day-to-day	0.21	16.9%	
Central Nervous System	Person-to-person	0.65	65.8%	0.99
	Month-to-month	0.14	14.4%	
	Day-to-day	0.20	19.8%	
Solid tumor	Person-to-person	0.84	76.8%	1.09
	Month-to-month	0.07	6.0%	
	Day-to-day	0.19	17.2%	
**All diagnoses**	Person-to-person	0.83	74.0%	1.12
	Month-to-month	0.09	8.3%	
	Day-to-day	0.20	17.7%	

Note: Hematological diagnoses include acute lymphoblastic leukemia, non-Hodgkin lymphoma, and Hodgkin lymphoma. Solid tumor diagnoses include Wilms tumor, neuroblastoma, rhabdomyosarcoma, osteosarcoma, and Ewing sarcoma.

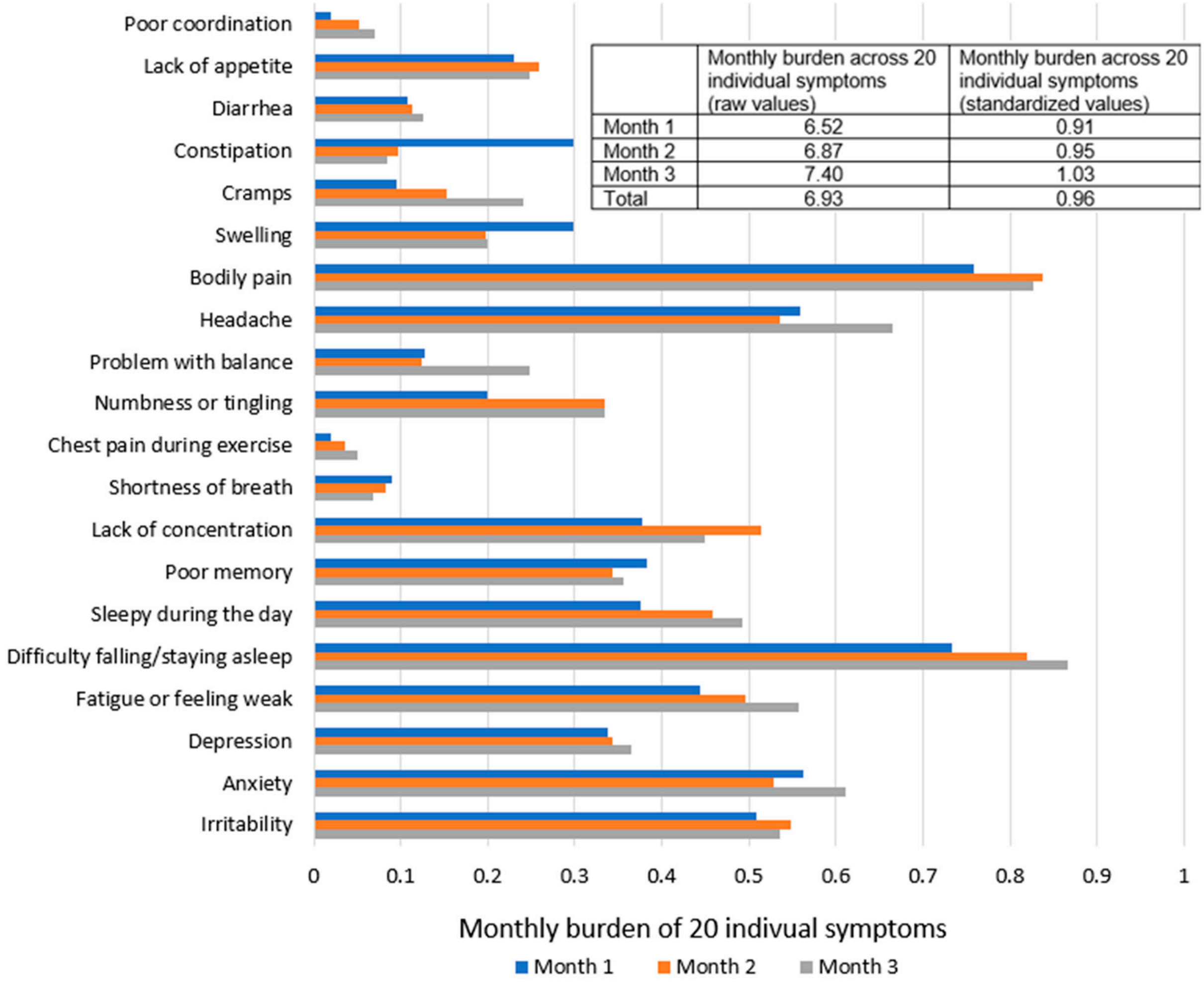

	Monthly burden across 20 individual symptoms (raw values)	Monthly burden across 20 individual symptoms (standardized values)
Month 1	6.52	0.91
Month 2	6.87	0.95
Month 3	7.40	1.03
Total	6.93	0.96

Supplemental Figure S1. Monthly burden of 20 individual symptoms (N=41)

Note: 1) Monthly burden of individual symptoms was defined as the mean of 5 daily symptom burdens for each of the three months (range: 0-3). 2) Monthly burden across 20 individual symptoms is the sum of the individual symptoms for each month (range: 0-60). 3) Total burden across 20 symptoms is the mean of symptom burdens over 3 months. 4) The standardized monthly burden is the sum of the monthly burden divided by the standard deviation of all monthly burdens (7.20).
